# Atomistic simulations suggest dietary flavonoids from Beta vulgaris (beet) as promising inhibitors of human angiotensin-converting enzyme and 2-alpha-adrenergic receptors in hypertension

**DOI:** 10.1093/bioadv/vbad133

**Published:** 2023-09-22

**Authors:** Joy A Adetunji, Oludare M Ogunyemi, Gideon A Gyebi, Anuoluwapo E Adewumi, Charles O Olaiya

**Affiliations:** Nutritional and Industrial Biochemistry Laboratory, Department of Biochemistry, Faculty of Basic Medical Sciences, College of Medicine, University of Ibadan, Ibadan 200005, Nigeria; Nutritional and Industrial Biochemistry Laboratory, Department of Biochemistry, Faculty of Basic Medical Sciences, College of Medicine, University of Ibadan, Ibadan 200005, Nigeria; Department of Biochemistry, Faculty of Science and Technology, Bingham University, Karu, Nigeria; Natural Products and Structural (Bio-Chem)-informatics Research Laboratory (NpsBC-Rl), Bingham University, Nasarawa, Nigeria; Nutritional and Industrial Biochemistry Laboratory, Department of Biochemistry, Faculty of Basic Medical Sciences, College of Medicine, University of Ibadan, Ibadan 200005, Nigeria; Nutritional and Industrial Biochemistry Laboratory, Department of Biochemistry, Faculty of Basic Medical Sciences, College of Medicine, University of Ibadan, Ibadan 200005, Nigeria

## Abstract

**Motivation:**

*Beta vulgaris* (beet) is extensively reported for its antihypertensive activity. However, the mechanismunderpinning its antihypertensive activity is not well understood. In this study, we evaluated the in silico interactionsof 70 compounds derived from beta vulgaris against the active sites of angiotensin-converting enzyme (ACE) and alpha-adrenergic receptor (AR).

**Results:**

Structure-based virtual screening against angiotensin-converting enzyme revealed that, Cochliophilin A (−9.0 Kcal/mol), Miraxanthin (−8.3 Kcal/mol), and quercimeritrin (−9.7 Kcal/mol) had lower docking scores than the reference lisinopril (−7.9 Kcal/mol). These compounds exhibited dual binding tendency as they also ranked top compounds upon screening against adrenergic receptor. The thermodynamic parameters computed from the resulting trajectories obtained from the 100 ns full atomistic molecular dynamics simulation revealed structural stability and conformational flexibility of the ligand–receptor complexes as indicated by the RMSD, RMSF, RoG, SASA, and H-bond calculations. The molecular mechanics with generalized Born and surface area solvation binding energy calculations revealed that the proteins exhibit considerable binding energy with the phytochemicals in a dynamic environment. Furthermore, the hit compounds possess good physicochemical properties and drug-likeness. Overall, cochliophilin and quercimeritrin are promising dual-target directed flavonoids from *Beta vulgaris*; and are suggested for further experimental and preclinical evaluation.

**Availability and implementation:**

All data was provided in the manuscript.

## 1 Introduction

Hypertension is the leading cause of cardiovascular disease-related death worldwide. The disease burden and prevalence of hypertension are significant worldwide. Currently, Africa has the highest disease burden and prevalence in the world with 46% of adults 25 years and above being hypertensive and there is a 30% estimated increase by the year 2025 (Ferdinand 2020, Sharma *et al.* 2021). Hypertension is a major public health concern with a 30% estimated increase by the year 2025 (Sharma *et al.* 2021). Despite the widespread use of antihypertensive drugs, it has not significantly impacted the global mean pressure. The prevalence has however increased with an increasing premature death toll, indicating the need for more research on effective pharmacological intervention in the management and treatment of hypertension (Mills *et al.* 2020, Zhou *et al.* 2021). Hypertension is the persistent elevation of the blood pressure in the systemic arteries, characterized by systolic blood pressure (SBP) of 140 mmHg or more and diastolic blood pressure (DBP) of 90 mmHg or more (Carey *et al.* 2018). The renin–angiotensin–aldosterone system (RAAS) is a critical regulator of blood volume and systemic vascular resistance, while the baroreceptor reflex responds in a short-term manner to decreased arterial pressure, making the RAAS a target for pharmacological therapy (Hall *et al.* 2021). The choice of an antihypertensive drug is based on individual tolerability and efficacy, however, angiotensin-converting enzyme (ACE) and angiotensin II receptor blockers are considered first-line medication for hypertension (Oparil *et al.* 2018). ACE inhibitors exert their effect on ACE by forming a zinc ligand, the functional group binding to ACE via the zinc moiety is the primary structural difference among these agents (Song and White 2002). Lisinopril and Captopril are the only ACE inhibitors that are not prodrugs, making them better treatment options for patients with severe hepatic disorder. The physiochemical characteristics of ACE inhibitors help determine at least part of their pharmacologic potency. After Angiotensin I is converted to Angiotensin II by ACE, it has effects on the kidney, adrenal cortex, arterioles, and brain by binding to Angiotensin II type I (AT) and type II (AT) receptors. The role of AT receptors is still being investigated, but pertinently, they have been shown to cause vasodilation by nitric oxide generation. In the plasma, angiotensin II has a half-life of 1–2 min, at which point peptidases degrade it into Angiotensins III and IV (Hall *et al.* 2019, Fountain and Lappin 2021). Adrenergic receptors (ARs) form the interface between the endogenous catecholamines epinephrine and norepinephrine and a wide array of target cells in the body to mediate the biological effects of the sympathetic nervous system (Philipp *et al.* 2002). 2-alpha-receptors are involved in the control of blood pressure homeostasis at several locations. The 2A-receptor is a therapeutic target for subtype-selective antihypertensive agents and is also required for the development of salt-sensitive hypertension (Philipp *et al.* 2002). Dexmedetomidine has been demonstrated to be a highly selective alpha2-adrenergic agonist. It reduces both heart rate and the mean arterial blood pressure (Bari *et al.* 1993). The most important active site residue for ACE includes GLU 162 HIS 353 ALA 354ASP 377 GLU 384HIS 387 LYS 511 HIS 513 TYR 520 and TYR 523 (Akif *et al.* 2011). The orthosteric pocket of alpha-AR includes ASP113, SER144, TRP182, PHE186, PHE189, HIS230, SER284, and PHE289 (Yuan *et al.* 2020). Inhibition of these proteins via interaction with target amino acid residues results in a reduction in blood pressure.

*Beta vulgaris* (beet) is a plant in the Amaranthaceae family sometimes called beetroot or garden beet (Kavitha *et al.* 2016). Beetroot is grown for food and likewise for sugar production and biofuel. It is also consumed as dietary supplement and has gradually become a functional food source (Liliana and Oana-Viorela 2020). In the western part of Nigeria, beetroot is commonly referred to as A*lubosa eleje*, *Isu dandan*, or *Koba-kogbe* by the Yorubas. Different classes of phytochemicals are reported to be present in beetroot, such as alkaloids, steroids, tannins, flavonoids, glycosides, saponins, and steroids (Afiomah and Iwuozor 2020), and are responsible for the pharmacological properties of beetroot (Bavec *et al.* 2010, Adetunji *et al.* 2023). Studies by Clifford *et al.* provided compelling evidence that beetroot ingestion offers beneficial physiological effects that may translate to improved clinical outcomes for several pathologies, such as; hypertension, and atherosclerosis (Clifford *et al.* 2015). In another study, Siervo *et al.* gave an account of the significant drop in SBP and DBP after doses of beetroot supplement, suggesting a dose–dependent basis for lowering blood pressure figures (Siervo *et al.* 2013). More recently, Alshehry *et al.* reported that beetroot has strong antihypertensive properties, although the specific phytochemical that mediates the decrease in blood pressure is not known yet (Alshehry *et al.* 2021). Computational analysis tools have been explored for phytochemical research aimed at drug design and discovery. Molecular docking of compounds is now being applied in medicinal chemistry, drug discovery and design, and structural molecular biology (Anifowose *et al.* 2023, Ogunyemi *et al.* 2023). The computer-assisted drug design CADD has been an efficient tool in the pharmaceutical industry, improving drug synthesis (Zhang 2011, Aldridge *et al.* 2013). Fatima *et al.* recently reported a computational-based approach that has been used in identifying a potential drug candidate against hypertension (Fatima *et al.* 2022). Crizotinib is another example of a successful drug demonstrated to be clinically effective that was developed using structure-based approach (Cui *et al.* 2020). Despite the popular dietary and ethnopharmacological use of beet in hypertension, most of the phytocompounds reported from this plant have not been explored for their role in alleviating hypertension. Therefore, this study employs computational methods to screen and assess the molecular interactions of 70 previously reported phytocompounds from beet with the active sites of human ACE and alpha-AR; to investigate and predict possible hit compounds and interactions between ligands (phytochemicals) and macromolecules (ACE and 2AR) using computational tools.

## 2 Methods

### 2.1 Ligand preparation

The phytochemical constituents of beet were obtained from literature containing experimental data on GCMS and x-ray crystallography on beetroot extracts. About 70 phytochemicals were compiled (Duke 1992, Reuss 2000). The 2D structures of the compounds and reference drugs were downloaded in Structure data file (SDF) format from PubChem (www.pubchem.ncbi.nlm.nih.gov), respectively. They were prepared to PDB format using the BIOVIA Discovery studio, uploaded to PyRx software and converted to dockable PDBQT format using the Open Babel plugin (O’Boyle *et al.* 2011) of the PyRx software. The output files were minimized to obtain the minimum energy for the ligand docking.

### 2.2 Protein preparation

The crystal 3D structure of Human ACE in complex with phosphonic tripeptide (PDB ID: 2XY9) as elucidated through X-ray diffraction as 1.97 Å resolution was downloaded from (https://www.rcsb.org/) with the protein database ID: 2XY9 (Akif *et al.* 2011). The structure of alpha 2-B adrenoceptor protein elucidated through electron microscopy with 4.1 Å resolution (PDB ID: 6K42) was also downloaded for the same web database (Yuan *et al.* 2020). After retrieval, the native crystallized ligands were extracted, and water molecules were removed from the structure. Hydrogen atoms were added to the structures using Autodock version 4.2 programs (Scripps Research Institute, La Jolla, CA).

### 2.3 Receptor grid box generation

The docking carried out was site-directed, done by selecting specific amino acids using the AutoDock plugin of the PyRx. The receptor grid file in Table 1 was generated using a receptor grid generation panel, which represents the active sites of the receptor for glide ligand docking jobs.

**Table 1.** Grid box parameters of ACE and adrenoceptor.

Dimensions	ACE (Å)	AR (Å)
Center_x	13.49	168.38
Center_y	5.80	165.80
Center_z	23.15	197.06
Size x	23.03	18.21
Size y	23.03	17.75
Size z	17.19	24.06
Exhaustiveness	8	8

### 2.4 Molecular docking simulations

**Figure 1.**
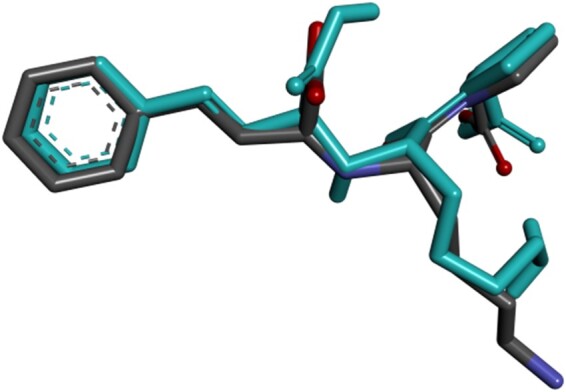
Superimposition of selected docked conformer of co-crystallized myricetin on the retracted co-crystallized structure.

Prior to docking analysis, the docking protocol was validated by redocking the native inhibitor into the active region of the enzyme, lisinopril, the co-crystallized compound with the human ACE structure (PDB ID: 1o86) was retracted from the structure. The compound was subsequently redocked into the same domain of the enzyme structure. The binding pose with the minimal binding energy was superimposed on the retrieved co-crystallized inhibitor (Fig. 1), after which the root mean square deviation (RMSD) was calculated using Discovery Studio. Molecular docking of the prepared ligands and proteins was performed using AutoDock Vina in the PyRx workspace. The ligand structures ligands were imported into AutoDock Vina in PyRx 0.8 and minimized using the incorporated Open Babel plugin applying the Universal Force Field as the energy minimization parameter and conjugate gradient descent as the optimization algorithm. The ligand structures were then screened against active sites of ACE and AR. The molecular docking simulations were initiated keeping all other parameters as default. The docking tools were set to generate eight poses for each of the ligands to be docked to the protein binding site. After docking simulation with ACE, the ligand poses with lower than −7.9 docking scores were computed and docked against AR for multi-target binding analysis. The molecular interactions were viewed with BIOVIA Discovery Studio (Dassault Systèmes, Visualizer version 21.1.0.0).

### 2.5 Molecular dynamics simulation

The complexes of ACE and AR with the two lead phytocompounds each from the docking experiment (Cochliophilin A and Quercimeritrin for ACE; Cochliophilin A and Miraxanthin III for AR) as well as those of the reference inhibitors, Lisinopril and Dexmedetomidine, respectively, were selected for molecular dynamics simulation (MDS) using Gromacs. The backbone and protein–ligand complexes were subjected to a 100 ns full atomistic MDS production run. The necessary MDS files were prepared using CHARMM-GUI and the CHARMM36 force field was used in the dynamic simulations (Jo *et al.* 2008, Brooks *et al.* 2009, Lee *et al.* 2016). The salt concentration and temperature of the biomolecular systems were set to 0.154 NaCl and 310 K, respectively, to mimic the physiological conditions. Before the production run, the system was minimized for 10 000 steps in a constant number of atoms, constant volume, and constant temperature (NVT) ensemble apply a conjugate gradient algorithm, and then equilibrated in a constant number of atoms, constant pressure, and constant temperature (NPT) ensemble for 1 ns as demonstrate earlier (Ogunyemi *et al.* 2023). The simulation pressure was set to 1.01325 bar and controlled by the Nose–Hoover Langevin piston, while the temperature was controlled by Langevin dynamics.

### 2.6 Free energy calculation

The binding free energy of each complex system was determined using the Molecular Mechanics Generalized Born Surface Area (MM-GBSA) method and decomposition analysis to get the binding energies of amino acids within 1 nm of the ligand as demonstrated earlier (Ogunyemi *et al.* 2023, Gyebi *et al.* 2023) using the gmx MMPBSA package (Miller *et al.* 2012, Valdés-Tresanco *et al.* 2021). The ionic strength was set to 0.154 M, and the solvation technique was set to 5. A value of 1.0 used for the internal dielectric constant and a value of 78.5 for the external dielectric constant, with all other parameters left in their default values. The MM-GBSA method is depicted in Equation (1)


(1) $$\Delta G=G_{\text{complex}}-G_{\text{receptor}}-G_{\text{ligand}}.$$

Different energy terms were calculated according to Equations (2)–(6)


(2) $$\Delta G_{\text{binding}}=\Delta H-T\Delta S,$$

(3) $$\Delta H=\Delta E_{\text{gas}}+\Delta E_{\text{sol}},$$

(4) $$\Delta E\text{gas}=\Delta E_{\text{ele}}+\Delta E_{\text{vdW}},$$

(5) $$\Delta E_{\text{solv}}=E_{\text{GB}}+E_{\text{SA}},$$

(6) $$E_{\text{SA}}=\gamma ._{\text{SASA}},$$

where:

Δ*H* is the calculated enthalpy from solvation-free energy (*E*_sol_) and gas-phase energy (*E*_gas_).

The *T*Δ*S* is the entropic contribution to the free binding energy but was not computed in this study.

*E*_gas_ comprises of van der Waals (EvdW) and electrostatic (*E*_ele_) terms.

*E*_sol_ was computed from the polar solvation energy (EGB) while (ESA) non-polar solvation energy was assessed from the accessible solvent surface area (Xue *et al.* 2018, Tuccinardi 2021).

### 2.7 ADMET studies

**Figure 2.**
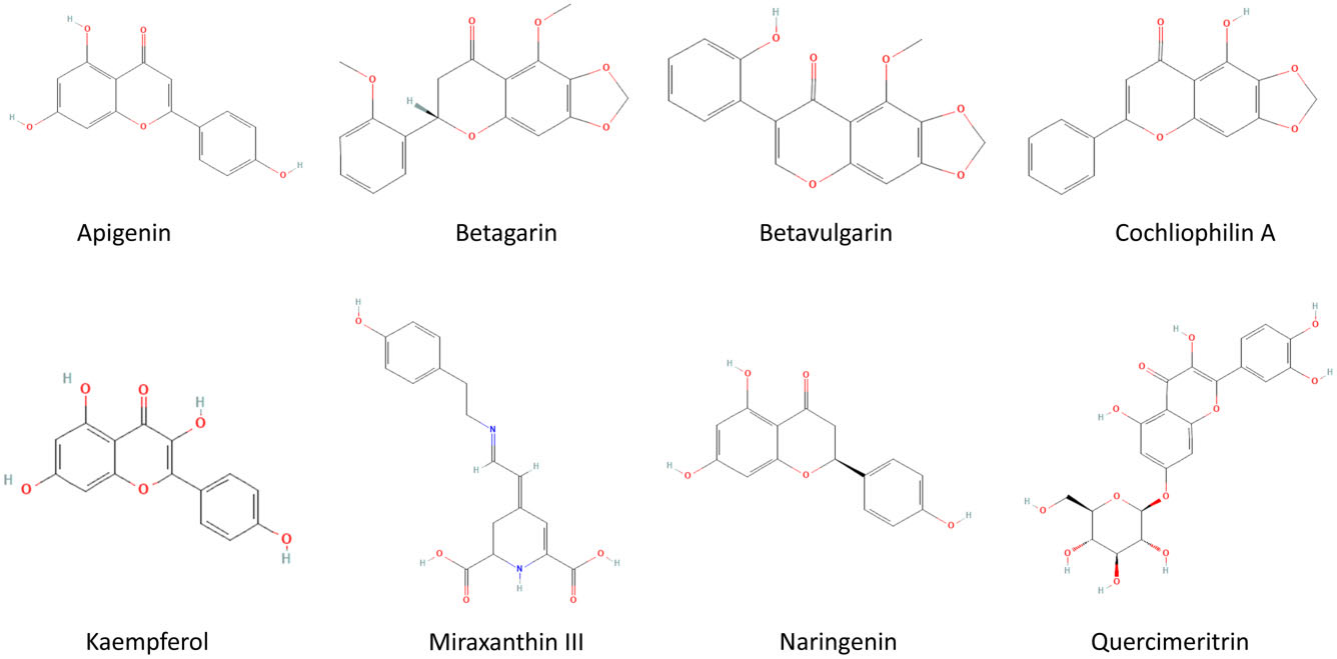
Top docking phytocompounds with target enzymes.

The ADMET properties including lipophilicity (log Po/w), water solubility (log S), drug-likeness, bioavailability score, pharmacokinetics, and toxicity profile of the test compounds were determined for the top eight compounds (Fig. 2) using *in silico* integrative model predictions at the SwissADME (http://www.swissadme.ch/index.php) (Daina *et al.* 2017) and PROTOX II (https://toxnew.charite.de/protox_II/index.php) (Banerjee *et al.* 2018) online servers. Parameters, such as Lipinski’s rule of five, were evaluated to predict the drug-likeness of the chemical compounds. The SDF files and canonical SMILES of the compounds were retrieved from the PubChem Database to estimate the ADMET properties using default parameters.

## 3 Results

### 3.1 Molecular docking studies

Molecular docking is of great importance in the planning and design of new drugs. It correctly predicts the experimental binding mode and affinity of a native molecule within the binding site of the drug target. A major, metabolic step in the pathophysiology of hypertension is the reaction catalyzed by ACE. In this study, selected compounds were investigated as potential inhibitors against ACE and alpha-AR. Human ACE was employed as one of the drug targets to evaluate its interaction with novel inhibitors alongside its reference drugs, lisinopril, and captopril.

To assess the performance of the docking protocol, which was based on the scoring function in AutoDock Vina, lisinopril, the co-crystallized compound with the human ACE structure (PDB ID: 1086) was retracted from the structure. The compound was subsequently redocked into the same domain of the enzyme structure. The result revealed that all the docking conformations of lisinopril structure were located within the active site region of the enzyme (Fig. 1). Estimation of the RMSD between the lowest binding pose and the initial crystal structure gave 2.50 Å, while the best-docked conformation of lisinopril with ACE had a docking score of −8.4 Kcal/mol.

This study revealed that, several phytochemical structures reported from beet exhibited lower binding energy than the reference antihypertensive drugs as shown in Table 2.

**Table 2.** Docking scores of lisinopril, captopril, dexmedetomidine, and top 10 hit compounds from beet against active site residues of ACE and AR.

S/N	Phytochemical	Class	Pubchem ID	ACE	AR
S1	Lisinopril			−7.9	
S2	Captopril			−6.0	
S3	Dexmedetomidine				−7.4
1	Quercimeritrin	Flavonoids	5 282 160	−9.7	−8.8
2	Beta-sitosterol	Steroid	222 284	−9.3	−8.8
3	2'-O-beta-D-Glucosylisovitexin	Flavonoids	185 995	−9.2	−7.7
4	Cochliophilin A	Flavonoids	927 642	−9.0	−9.1
5	Betagarin	Flavonoids	442 261	−8.9	−7.4
6	Betavulgarin	Isoflavonol	442 668	−8.7	−7.7
7	Miraxanthin III	Carboxylic acid	135 935 708	−8.3	−8.5
8	Kaempferol	Flavonoids	5 280 863	−8.1	−8
9	Apigenin	Flavonoids	5 280 443	−8.0	−7.9
10	Naringenin	Flavonoids	932	−8.0	−8.1

Lisinopril and captopril, the reference inhibitors have binding affinities of −7.9 and −6 Kcal/mol for ACE, respectively. Ranking based on the negative and low value of DG, and comparing with the reference inhibitors, presented docking scores ranging from −10.3 to −1.6 Kcal/mol. The docking analysis of the phytochemicals in comparison with the reference drugs (lisinopril and captopril) against the active site of the ACE shows that the compounds exhibited good docking conformations. Subsequent docking with the active region of the alpha-AR revealed 22 compounds with binding affinities (−9.1 to −7.4 Kcal/mol) lower than the reference drug Dexmedetomidine (−7.4 Kcal/mol). These compounds are presented in Table 2.

### 3.2 Molecular interaction of hit compounds with enzyme/receptor

The lead phytocompounds for each enzyme and receptor target were selected for interaction analysis based on their docking scores and interactions with active regions of the receptors in comparison with the reference compounds. The interactions involved hydrogen bond interaction, hydrophobic interactions, and other interactions. Assessment of the interactions showed that the phytocompounds and the reference drugs elicited good interactions including several active site residues of ACE as shown in Table 3.

**Table 3.** Interacting amino acid residue of the target enzyme and receptor with the top binding phytocompounds from beet.

Compounds	Enzymes	Hydrogen bonds interactions (bond distance Å)		Hydrophobic interaction		Other interactions	
		Numbers	Residues	Numbers	Residues	Numbers	Residues
Cochliophilin A	ACE	4	GLN^281^ (2.85); HIS^513^ (2.19, 2.69); HIS^353^ (2.34)	7	TYR^523^ PHE^527^ HIS^353^ TYR^523^ HIS^383^ TYR^523^ HIS^513^	0	None
Lisinopril		6	ALA^354^ (2.11, 2.44); HIS^513^ (2.09); ASP^415^ (2.26, 2.97); GLU^162^ (2.42)	5	PHE^457^ TYR^523^ VAL^380^ HIS^353^ HIS^383^	0	None
Betavulgarin		3	HIS^383^ (2.2) HIS^387^ (2.24, 2.88)	5	HIS^383^ HIS^387^ ALA^356^ VAL^518^	1	HIS^383^
Quercimeritrin		9	HIS^387^ (2.11); GLU^384^ (2.58); TYR^520^ (2.72); GLN^281^ (2.06, 2.64); LYS^511^ (2.93, 2.78); HIS^513^ (2.89); GLU^162^ (2.89)	1	VAL^380^	3	HIS^353^ GLU^162^ HIS^353^
Betagarin	AR	5	HIS ^387^ (2.24, 2.88); HIS ^383^ (2.19); HIS^353^ (2.10); HIS^513^ (2.23)	6	VAL^380^ HIS^353^ TYR^523^ ALA^354^ HIS^387^ VAL^518^	0	None
Naringenin		6	CYS ^370^ (2.95); GLU^162^ (2.35); GLN^281^ (2.15); LYS^511^ (2.46, 1.97, 2.66); TYR^520^ (2.21)	3	VAL^380^ TYR^523^ ALA^354^	4	HIS^353^ ASP^377^ GLU^162^ HIS^513^
Cochliophilin A		2	SER^180^ (2.92); SER^176^ (1.94)	7	LEU^166^ VAL^93^ PHE^412^ VAL^93^ CYS^96^ VAL^93^ TYR^391^ CYS^96^	0	None
Dexmedetomidine		1	ASP^92^ (2.20)	9	TYR^391^ LEU^166^ PHE^388^ TYR^391^ VAL^93^ PHE^388^ VAL^93^ LEU^166^ PHE^387^	0	None
Miraxanthin III	AR	5	ASN ^167^(2.49); GLN^168^ (2.58); ASP^92^ (2.51); SER^180^ (2.97); CYS^96^ (3.57)	3	LEU^166^ LEU^89^ PRO^147^	0	None
Quercimeritrin		3	SER^69^ (2.20); GLU^73^ (2.16); ASN^167^ (2.89)	1	LYS^165^	0	None
Naringenin		3	PHE ^412^ (1.16); SER^180^ (2.70) CYS^96^ (4.10)	2	VAL^93^ PHE^412^	0	None
Kaempferol		2	PHE ^412^ (2.23); SER^180^ (2.62)	6	VAL^93^ PHE^412^ VAL ^93^ CYS ^96^ VAL ^93^ CYS ^96^	0	None

**Table 4.** Eight hit compounds with multi-targeting potential.

Phytochemical	Bioavailability	Lipinski/drug-likeness	Toxicity class	LD50 (mg/kg)	Hepatotoxicity	Probability
Lisinopril	0.55	No violations	6	8500	Inactive	0.93
Captopril	0.56	No violations	5	2078	Inactive	0.89
Dexmedetomidine	0.55	No violations	3	155	Inactive	0.85
Apigenin	0.55	No violations	5	2500	Inactive	0.68
Betavulgarin	0.55	No violations	5	2500	Inactive	0.82
Betagarin	0.55	No violations	4	2000	Inactive	0.78
Cochliophilin A	0.55	No violations	5	4000	Inactive	0.78
Kaempferol	0.55	No violations	5	3919	Inactive	0.68
Miraxanthin III	0.56	No violations	4	750	Inactive	0.75
Naringenin	0.55	No violations	4	2000	Inactive	0.67
Quercimeritrin	0.11	No violations	5	5000	Inactive	0.82

Quercimeritrin shows similar bind interaction with the reference drugs, forming a hydrogen bond with HIS^513^ and GLU^162^ and hydrophobic interactions with HIS^353^ and GLU^162^. Cochliophilin A in a similar pattern to the reference drug forms hydrogen interactions with HIS^353^ and hydrophobic interactions with TYR^523^ and HIS^513^. Likewise, the Cochliophilin A and Miraxanthin III and reference drugs showed good interactions with the orthosteric pocket of AR forming hydrogen bonds with the active site amino acids; ASP^92^, SER^180^, and hydrophobic interactions with the orthosteric pocket TYR^391^ and LEU^166^.

Protein–ligand complexes play a pivotal role in biological systems and the environment. The ligands can be an inhibitor, signal transducer, cofactor, allosteric regulator, and activator. The role helps to define protein function while the structure of these complexes aids the analysis of the interaction between the ligand and the protein. The detailed analysis of the hydrogen bond formed is crucial in studying their interaction (Rajan *et al.* 2013).

**Figure 3.**
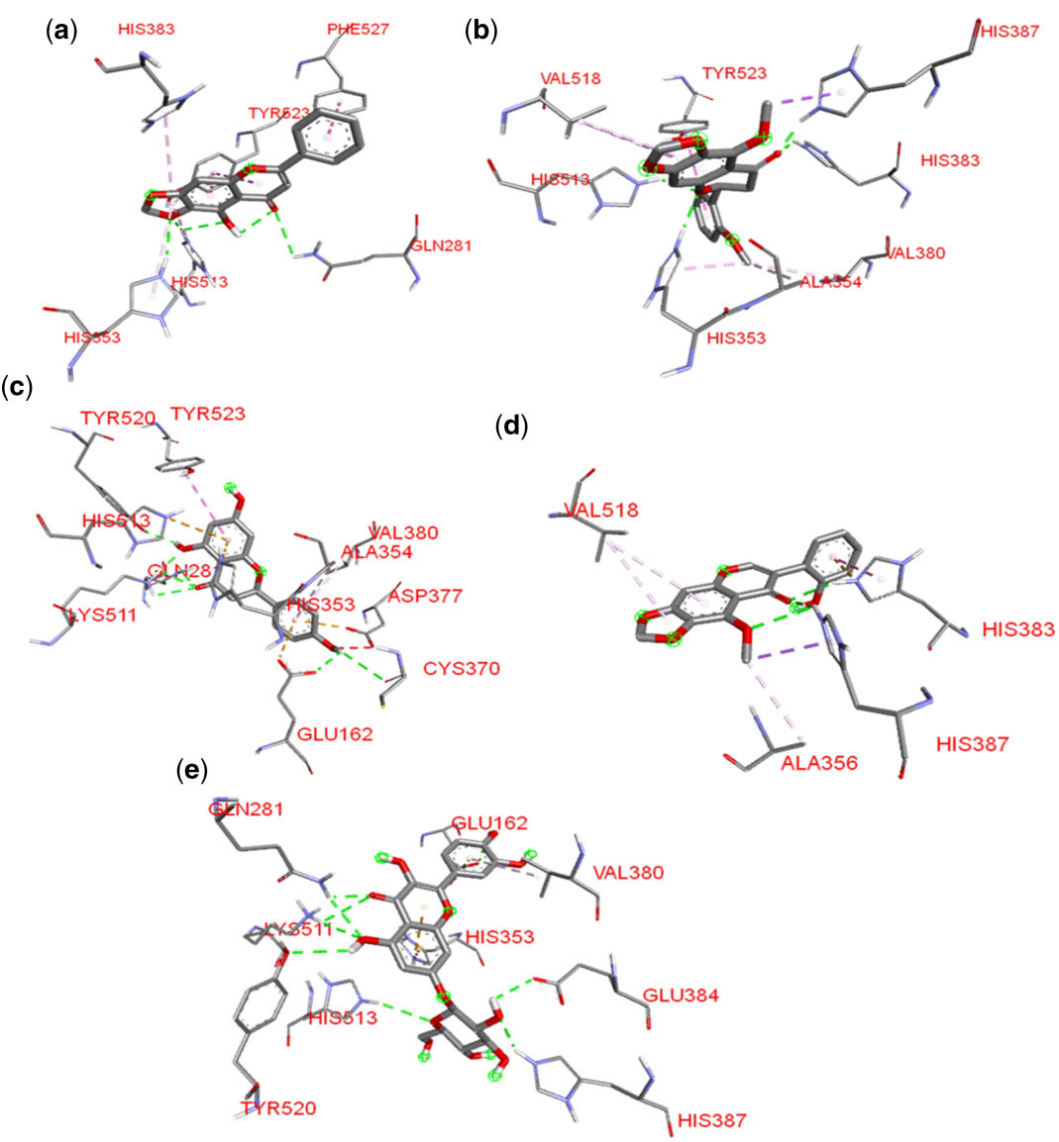
3D representation of the amino acid interactions of the ACE with (a) Cochliophilin A, (b) Betagarin, (c) Naringenin, (d) Betavulgarin, and (e) Quercimeritrin amino acids are represented by 3-letter abbreviations. Hydrogen bonds, hydrophobic interactions, and other interactions are represented by green, purple, and Liliac dotted lines, respectively.

Specific interaction analysis revealed that Cochliophilin A formed a complex with ACE via the hydrogen bonding with GLN^281^, HIS^513^, HIS^353^, and hydrophobic interactions with TYR^523^, PHE^527^, HIS^353^, HIS^383^, and HIS^513^ while Lisinopril elicited strong binding interactions to ACE with five hydrogen bonds including HIS^513^ and four hydrophobic interactions (Fig. 3).

The interactions were maintained by the benzene ring of Lisinopril. Cochliophilin A, the second hit phytocompound forms pi–pi T-shaped hydrophobic interaction PHE^527^. This bond involves the aromatic side chain of PHE^527^ and the phenyl B ring of the flavonoid (flavone). TYR^523^ is coordinated to the A and C rings of the benzopyran ring of the flavone backbone via pi–pi stacked hydrophobic interactions. This interaction is like the TYR^523^ and phenyl ring in Lisinopril. Pi–pi stacking is non-covalent attractive interaction that are vital in the organization of biomolecular structures and biological recognition occurring between benzene rings.

Comparably the hit phytocompound Quercimeritrin, a flavonoid glycoside displayed nine hydrogen bonding five of which were with the active site residues HIS^387^, TYR^520^, LYS^511^, HIS^513^, and GLU^162^. This phytocompound formed pi–anion interaction between its phenyl rings and the side chain of GLU^162^. The benzopyran ring likewise forms electrostatic interaction via pi–cation and the side residue of HIS^353^. The phenyl B ring of the flavonoid backbone forms pi-alkyl hydrophobic interaction with VAL^380^. The interaction profile of Quercimeritrin is like that of lisinopril, forming hydrogen bonds with HIS^513^ (2.89); GLU^162^.

Betagarin belonging to the flavanone class of flavonoids maintains conventional hydrogen bonds with HIS^387^, HIS^383^, HIS^353^, and HIS^513^; pi–pi T-shaped hydrophobic interaction with TYR^523^; pi–sigma interaction with HIS^387^; alkyl interaction with ALA^354^ and VAL^518^ and pi-alkyl with HIS^353^ and VAL^518^ in comparison to lisinopril, betagarin similar hydrogen bonding, and hydrophobic interaction. Naringenin, a flavan interacts with ACE forming six hydrogen bonds inclusive of active site amino acids GLU^162^, LYS^511^, and TYR^520^: pi–cation electrostatic interaction with HIS^383^, and pi–anion interaction with ALA^356^ and VAL^518^.

It also forms hydrophobic pi–pi T-shaped and pi-alkyl interactions with target amino acids. Betavulgarin had three hydrogen bond interactions with HIS^383^ and HIS^387^; electrostatic pi–cation interaction with HIS^383^, hydrophobic pi–sigma bond with HIS^387^, pi–pi stacked interaction with HIS^383^, pi-alkyl interaction with VAL^518^, and alkyl interactions with ALA^356^ and VAL^518^.

**Figure 4.**
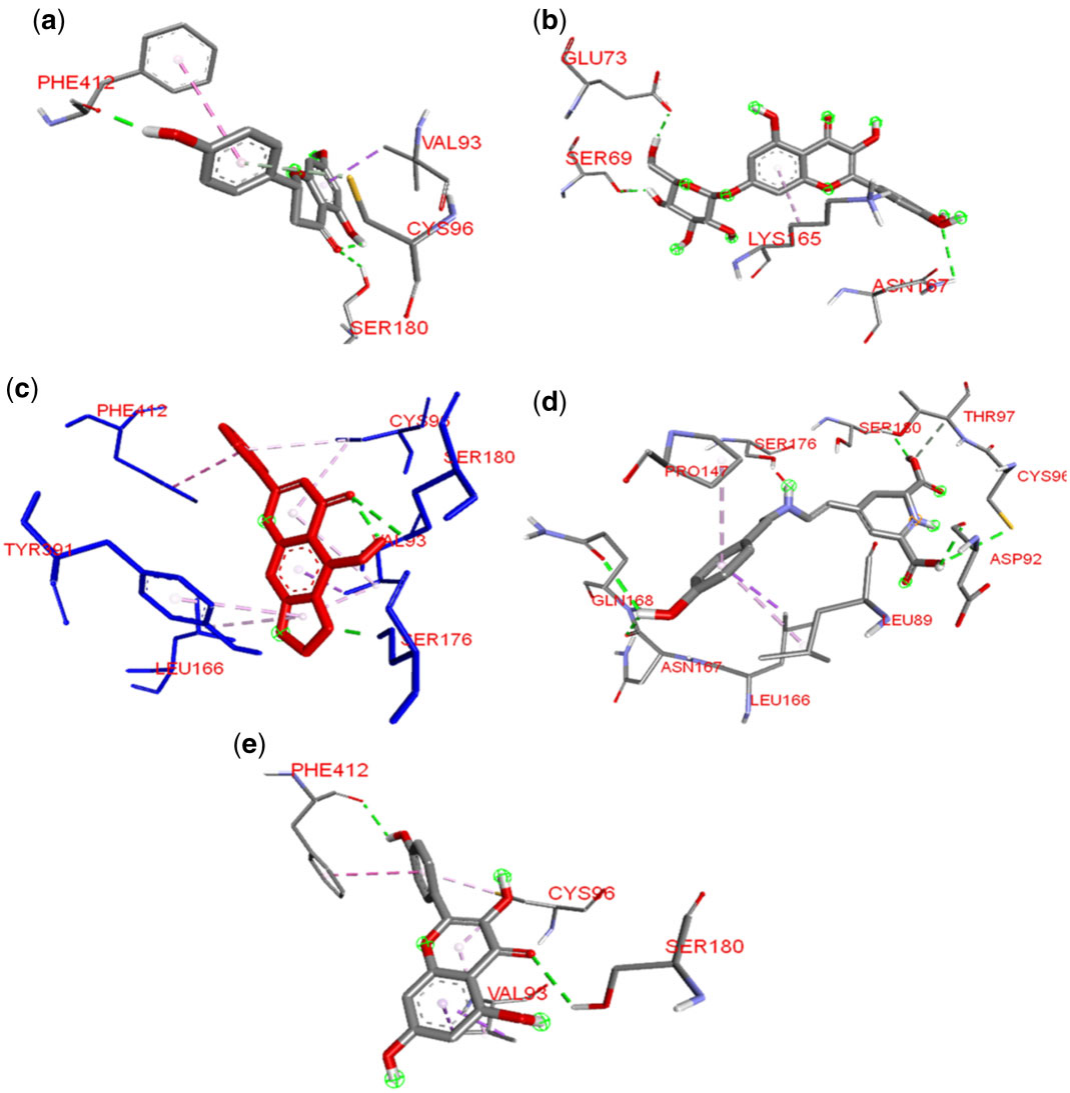
3D representation of the amino acid interactions of the 2-α AR with (a) Cochliophilin A, (b) Kaempferol, (c) Miraxanthin III, (d) Naringenin, and (e) Quercimeritrin. Amino acids are represented by three-letter abbreviations. Hydrogen bonds, hydrophobic interactions, and other interactions are represented by green, purple, and Liliac dotted lines, respectively.

The beetroot-derived compounds were docked against the active site region of AR, Fig. 4 shows the 3D representation of the amino acid interaction of AR with the phytocompounds. Cochliophilin A, the top docked compound with AR after the previous docking with ACE demonstrated a higher binding affinity. The flavonoid demonstrates the lowest binding energy with AR. Cochliophilin A interacts with the side chain of active site amino acid via the oxygen of the benzopyranone rings (A and C rings). The oxygen for the 2-phenylchromen-4-one accepts hydrogen from the serine side chains forming strong interactions.

Furthermore, this phytocompound forms seven hydrophobic interactions with amino acids that make up the orthosteric pocket of AR. A pi–sigma interaction with VAL^93^, pi–pi stacked interaction with PHE^412^, two alkyl interactions with LEU^166^ VAL^93^, and four pi-alkyl interactions with VAL^93^, CYS^96^, and TYR^391^.

Miraxanthin III the second top hit compound interacted with AR forming five conventional hydrogen binds, three of which are active site residues. It also forms two pi-alkyl hydrophobic interactions with LEU^89^ and PRO^147^ and one pi–sigma hydrophobic interaction with LEU^166^ an active site residue. Kaempferol; a flavonol (hydroxy flavone) forms a conventional hydrogen bond with PHE ^412^ and SER^180^ via the oxygen of the phenyl B ring and the ketone oxygen of the C ring, respectively. Pi–pi stacked and pi–sigma and pi-alkyl interactions are all maintained with PHE^412^, VAL^93^, and CYS^96^. Observations from these molecular interactions provide mechanistic insight into *in vitro* and *in vivo* reports on the blood pressure-lowering activity of beetroot. A recent study (Baião *et al.* 2020) and a current review (Liliana and Oana-Viorela 2020) on the antihypertensive property of beetroot/red beet juice indicates that phytochemical bioavailability in beetroot contributes to the vascular protective effect and blood-pressure-lowering/antihypertensive activity via inhibiting ACE. These studies agree with the 2-fold inhibitory potential of the flavonol (Quercimeritrin) and flavone (Cochliophilin A) on ACE and 2 alpha-ARs.

### 3.3 Molecular dynamics simulation

The recognition between a ligand (substrate or regulator) and a macromolecule is by nature a dynamic process. This process requires structural rearrangement (Hospital *et al.* 2015). Molecular docking provides or predicts the binding affinity between a macromolecule and a ligand. However, when a ligand approaches a protein e.g. a receptor, it encounters a macromolecule that is in constant dynamic motion. MDSs, therefore, provide insights into protein motion, which is significant in drug discovery (Durrant and McCammon 2011). MDS can be simply put as protein–ligand interaction at the atomic level, it gives an insight into what occurs *in vivo*. An atomic-level structure is vital and provides insightful information about how the biomolecule functions, conformational changes, ligand binding, and protein folding (Hollingsworth and Dror 2018). Assessment and evaluation of the stability of complexes and computation of thermodynamic parameters are mediated by MDSs. This validates the static model of ligand–protein interaction. In this study, 100 ns MDS was performed to compare the stability and structural conformation of the ligand–enzyme, ligand–receptor complexes with the unbound enzyme/receptor (apoprotein) in a full atomistic dynamic environment.

### 3.4 Trajectory analysis of ligand

#### 3.4.1 RMSD of atomic position

In structure-based drug design, the RMSD is a measure of the difference between a crystal conformation of the ligand conformation and a docking prediction. The stability of the native state (the apoprotein) is investigated with respect to the reference structure i.e. when bound to the ligand. It, therefore, measures the deviation from the overlap of compared structures. Measurements are based on the alpha carbon atoms (C_α_), which are the backbone of amino acids in proteins (Arnittali *et al.* 2019). The smaller the deviation the more spatially equivalent the two compared structures are. This also gives information about the extent of stability of the complex.

**Figure 5.**
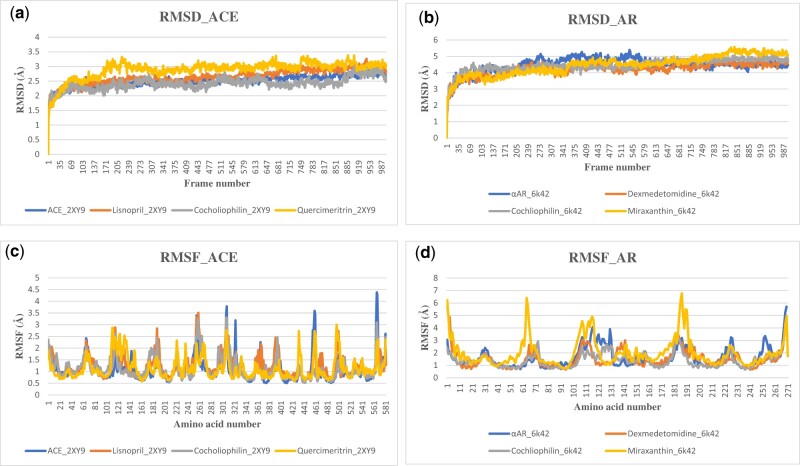
The backbone-RMSD plots of MDS of (a) ACE (b) 2-α AR. Per residue RMSF plots of MDS of (c) ACE (d) 2-α AR (apo and protein–ligands complexes).

The RMSD plot for the apoenzyme 2XY9 (ACE) and its complexes lisinopril, Quercimeritrin, and Cochliophilin A is shown in Fig. 5a the plot showed stable trajectories featuring “consistent” and minor fluctuations. This observation during MDS indicates that the biomolecular system is equilibrated and stabilized while higher fluctuations indicate lower stability. The apoprotein and the protein complex show an initial rise to 1.6 Å, where they are stabilized, and subsequent fluctuation is between 1.8 and 3.2 Å. At frame 169 (16.9 ns) 2XY9_Quercemaritrin shows a slight increase in fluctuation from 2.6 Å to a value of 3.3 Å at frame 219 (21.9 ns) and finally stabilizes at frames 250 (25 ns). However, since the fluctuations are within 2.0 Å and the trajectories are stable, the biomolecular system has obtained a stable conformation during the MDS without significant conformational transitions. The RMSD plot for Apoprotein 6K42, Dexmedetomidine, Cochliophilin A, and Miraxanthin III is indicated in Fig. 5b. The apoprotein shows a slight rise in the trend from 4.46 Å at 22.6 ns, the fluctuating rising trend continues to about 5.14 Å at 39.6 ns before stabilizing back to 4.46 Å at 65.1 ns. Generally, the RMSD plots show that the apoprotein and protein–ligand complex reaches a stable conformation during the MDS runs. The plot also indicates that although they have a similar binding pattern, the Cochliophilin A_6K42 complex had a more stable conformation during the MDS runs. 6K47_Cochliophilin A complex showed the steadiest RMDS tones across the 1000 frames (100 ns) all-atoms MDS.

#### 3.4.2 Root mean squared fluctuation

RMSD and root mean squared fluctuation (RMSF) are two common measures of structural fluctuations in molecular dynamics (MD) (Martínez 2015). RMSF plot computed from the MDS (Fig. 5) trajectory files was estimated for each of the protein–ligand complex relative to the apo-state. The RMSF plot for the apoprotein (2XY9), 2XY9_lisinopril, 2XY9_Cohliophilin, and 2XY9_Quercimeritrin are depicted in Fig. 5. RMSF estimates the time evolution of the average deviation for each residue from its reference position (Al-Karmalawy *et al.* 2021). RMSF validation parameter highlights the contribution of individual amino acid residue within protein–ligand complex stability (Elhady *et al.* 2021).

The dynamic behavior of the protein residue in terms of flexibility is evaluated by explaining the mean deviation of each protein residue relative to its reference position over time. The apoprotein, Cochliophilin A_2XY9, and Lisinopril_2XY9 show similar fluctuation patterns. The apoprotein (6K42) had fewer fluctuations compared to the 6K42_Dexmedetomidine (Fig. 5) complex suggesting the protein is less flexible with the bound ligand. However, the 6K42_Miraxanthin III complex had high fluctuations (RMSD) value for almost all the critical amino acid residues, thus, 6K42_Miraxanthin III had a more flexible nature and there were structural changes. This increased interaction indicates that the ligand may be able to adapt well to the binding pocket of the protein. Higher RMSF values indicated greater flexibility during MDS (Zhao *et al.* 2015), and for a receptor–ligand complex like the 6K42–ligand interaction in this study, it indicated a higher adaptation for accommodation and binding stability.

#### 3.4.3 The radius of gyration

**Figure 6.**
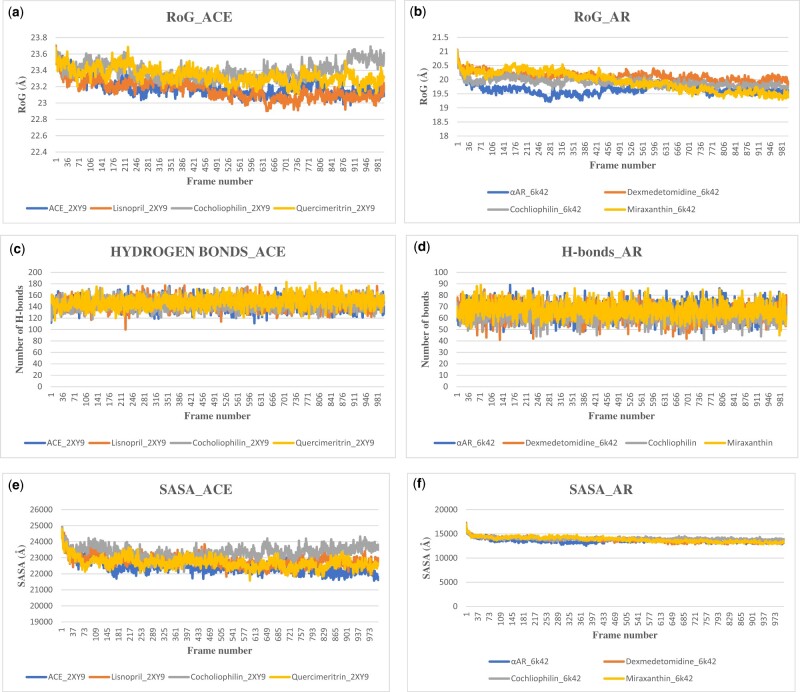
The RoG plots of MDS of (a) ACE (b) 2-α AR. The changes in the number of H-bonds during the MDS trajectory of (c) ACE (d) 2-α AR (apo and protein–ligands complexes). The SASA plots of MDS of (e) ACE (f) 2-α AR (apo and protein–ligands complexes).

To further elucidate the stability of the complex, the radii of gyration (RoGs) were monitored across the whole MD’ trajectories using GROMACS (gmx_gyrate) command script. RoG is the mass-weighted RMSD for a group of atoms relative to their common mass central (Al-Karmalawy *et al.* 2021), providing insight into their overall protein dimension. The stability parameter evaluated with RoG accounts for the global stability of either the ligand or tertiary structure of the protein. Taken together, a high RoG value achieving a plateau around an average value for the apoprotein and protein–ligand complexes in Fig. 6 depicts sustained stability or compactness of the biomolecular environment. Table 5 shows the mean values of the biomolecular systems.

**Table 5.** Mean RoG values of biomolecular systems.

Biomolecular system/complexes		Mean values (Å)
2XY9	ACE_2XY9	23.193
	Lisnopril_2XY9	23.159
	Cocholiophilin_2XY9	23.398
	Quercimeritrin_2XY9	23.335
6K42	αAR_6k42	19.645
	Dexmedetomidine_6k42	20.109
	Cochliophilin_6k42	19.911
	Miraxanthin_6k42	19.943

### 3.5 Binding interaction analysis

#### 3.5.1 H-bonds

H-bond interaction between the protein and the ligand is crucial for understanding the observed conformational changes and stability of the ligand–protein complex. H-bonds is a more detailed analysis at the atomic level performed through the calculation of the number of formed hydrogen bonds in all systems (Arnittali *et al.* 2019). Using the VMD hydrogen bonds tool, the established ligand–protein hydrogen bond interaction and their relative frequencies were explored. Hydrogen bonds provide most of the directional interactions that underlie protein structure, protein folding, and molecular recognition. Hydrogen bonds provide rigidity to the protein structure and specificity to intermolecular interactions. Observation of the four biomolecular systems shows a stable trend in the number of H-bonds, with an average of 145,145,150, and 152 for the apoprotein (2XY9), 2XY9_lisinopril, 2XY9_Cohliophilin, and 2XY9_Quercimeritrin, respectively (Fig. 6), aside from the drop in H-bonds for lisinopril to 100 at 225 frames (22.5 ns). The fluctuation of H-bonds is likewise stable for 6K42 and the complexes with an average of 74, 61, 60, and 67 H-bonds for apoprotein (6K42), 6K42_ Dexmedetomidine, 6K42_Cohliophilin, and 6K42_Miraxanthin III, respectively (Fig. 6).

#### 3.5.2 Solvent-accessible surface area

The solvent-accessible surface area (SASA) calculation addresses the surface properties of the peptide or protein. SASA accounts for the biomolecular surface area that is accessible to solvent molecules (Kamaraj and Purohit 2013) providing a quantitative measurement of the extent of a protein/solve interaction (Al-Karmalawy *et al.* 2021). The plotted SASA values are in alignment with the radius of the gyration trend of the simulated trajectories at 100 ns. The plot (Fig. 6e and f) showed that SASA is decreased after the binding of a ligand, the most obvious is the case of 6K42 and its ligand complexes. This suggests higher compactness ad stability of the docked complexes during simulation. The slight rise in the case of 2XY9_Cochliophilin A indicates an exposure of some buried residues to the solvent. However, it does not involve structural shifts. The stable values of SASA, RoG, and H-bonds indicate that the system was stable during the simulation time and not likely to undergo an unfolding process. This correlates with previous RMSD analysis confirming preferential better stability of Cochliophilin A and Quercimeritrin over lisinopril and Cochliophilin A and Miraxanthin III over dexmedetomidine.

### 3.6 Free energy calculation

Simulation-based estimation of free binding energy of ligands to proteins in a dynamic environment has been demonstrated to be a more considerably reliable and accurate calculations of the binding affinity using MM-GBSA as it accounts for solvation effects and entropy contributions to the binding (Perez *et al.* 2016, Ogunyemi *et al.* 2023). Thus, the ΔGbind computations provides an in-depth information about the binding modes of best-docked compounds at the initial stages of drug design and development (Kollman *et al.* 2000, Gyebi *et al.* 2023). The results for the ACE biomolecular system are shown in Table 6.

**Table 6.** The average binding free energy and the energy terms.

Energy terms (Kcal/mol)	Cocholiophilin_2XY9 complex	Quercimeritrin_2XY9 complex
Δ*E*_VDW_	−19.57 ± 4.81	−28.41 ± 5.10
Δ*E*_ELE_	−7.43 ± 3.74	−47.78 ± 13.23
Δ*G*_GB_	22.97 ± 10.27	61.39 ± 11.30
Δ*G*_SA_	−2.73 ± 0.78	−4.31 ± 0.06
Δ*G*_GAS_	−27.00 ± 13.39	−4.32 ± 0.69
Δ*G*_SOLV_	20.24 ± 9.80	57.07 ± 10.82
Δ*G*_TOTAL_	−6.76 ± 4.42	−19.12 ± 6.17

**Figure 7.**
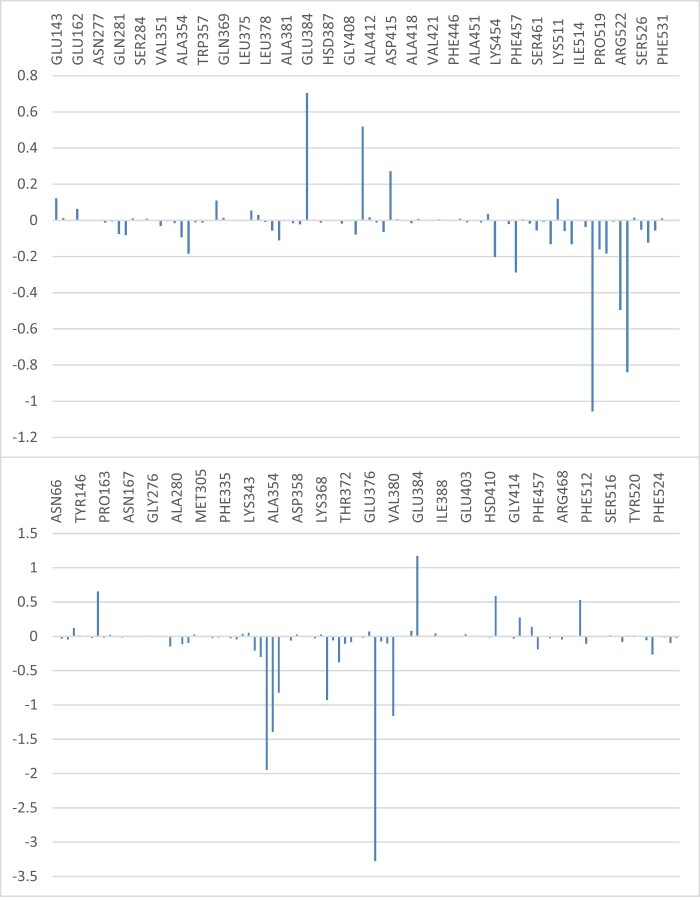
MM-GBSA plot of binding free energy contribution per residue of human ACE-phytochemical complexes (top) ACE-cocholiophilin (bottom) ACE-Quercimeritrin.

The results showed that quercimeritrin possessed considerable binding affinity (−19.12 ± 6.17 Kcal/mol) with ACE. This is higher than that of Cocholiophilin (−6.76 ± 4.42 Kcal/mol); and thereby validates the results of the molecular docking as reported in this study. The MM-GBSA binding energy of quercimetrin was mainly contributed by van der Waals (Δ*E*_VDW_) as shown in Table 6. The MMPBSA free energy decomposition of residues of the ligands in the protein–ligand complexes is depicted in Fig. 7.

Key residues around the binding site region of ACE contributed significantly to the overall binding free energy of the protein with the flavonoid structures based on the results of the MMPBSA free energy decomposition of residues of the ligands in the complexes (Fig. 7).

### 3.7 Phytochemical screening and ADMET analysis

ADMET analysis is a procedure used for defining whether the compounds can be easily absorbed, transported to their target site of action, metabolized in a way that still retains the pharmacological property, and is easily eliminated from the body while preventing toxic responses. These properties are generally termed ADMET (absorption, distribution, metabolism, elimination, and toxicity) (Sun *et al.* 2014). *In silico* prediction of ADMET properties is an alternative to standard experimental approaches (Ntie-Kang 2013), thus, has been an essential part of the drug discovery process (Darvas *et al.* 2002). This has helped in reducing the rate of pharmacokinetics-related failure of drugs in the clinical phases (Lombardo *et al.* 2003). Lipophilicity and water solubility are critical physicochemical properties that determine the ADMET behaviors of a drug. An orally administered drug should be sufficiently lipophilic to pass through the intestinal lining, penetrate the membrane of target cells and be sufficiently hydrophilic to travel in the aqueous blood. The higher the log *P*-value of a compound, the higher its lipophilicity and the lower its water solubility.

Drug-likeness analysis, a qualitative assessment of oral bioavailability is a feature of advanced stages of drug development (Daina *et al.* 2017). Tables 4 and 7 features the SwissADMET (http://www.swissadme.ch/index.php) (Daina *et al.* 2017) prediction of lipophilicity, drug-likeness, water solubility, and physicochemical and bioavailability scores of the hit compounds. Christopher A. Lipinski (1997) formulated the Lipinski rule of five. The rule describes the physicochemical properties important for a drug’s pharmacokinetics in the human body composing their absorption, distribution, metabolism, and excretion (Lipinski *et al.* 1997). However, the rule does not predict the pharmacological activity of the compound. The rule states that for a drug to be orally bioavailable it should have; a molecular weight ≤500 g/mol, log *P* (octanol-water partition coefficient) ≤5, hydrogen bond acceptors ≤10, and hydrogen bond donors ≤5 (Lipinski *et al.* 1997).

**Table 7.** ADMET, physicochemical, and drug-likeness properties of multi-target binding phytochemicals of beet.

Descriptors	Cochliophilin A	Quercemetrin	Miraxanthin III
Absorption			
HIA			
GI absorption	High	Low	High
Log *K*_p_ (skin permeation) cm/s	−5.10	−8.88	−7.32
P-glycoprotein substrate	No	No	No
P-glycoprotein inhibitor			
Distribution			
Blood–brain barrier	Yes	No	No
Metabolism			
CYP450 1A2 inhibitor	Yes	No	No
CYP450 3A4 inhibitor	Yes	No	No
CYP4502C9 inhibitor	Yes	No	No
CYP4502C19 inhibitor	No	No	No
CYP4502D6 inhibitor	Yes	No	No
Toxicity			
hERG blockers			
Hepatotoxicity	Inactive (0.78)	Inactive (0.75)	Inactive (0.82)
Carcinogenesis	Active (0.68)	Inactive (0.61)	Inactive (0.85)
Immunotoxicity	Inactive (0.79)	Inactive (0.94)	Active (0.58)
Mutagenicity	Inactive (0.61)	Inactive (0.69)	Inactive (0.76)
Cytotoxicity	Inactive (0.82)	Inactive (0.66)	Inactive (0.69)
PAINS	0 alert	1 alert	0 alert
Mitochondrial membrane potential	Inactive (0.59)	Inactive (0.83)	Inactive (0.98)
Physiochemical properties			
Molecular weight (g/mol)	282.25	464.38	330.34
Num. heavy atoms	21	33	24
Num. aromatic heavy atoms	16	16	6
Num. rotatable bonds	1	4	6
Num. H-bond acceptors	5	12	6
Hydrogen bond donor	1	8	4
iLogP	2.74	1.54	1.53
XLogP	4.11	0.36	1.4
WLogP	2.89	−0.54	0.97
MLogP	1.17	−2.59	−1.88
Molar refractivity	76.01	110.16	92.38
TPSA (Å²)	68.9	210.51	119.22
Druglikeness			
Lipinski	No violation	2 violations	No violation
Bioavailability score	0.55	0.17	0.56

The hit compounds elicit physiochemical properties that enable them to be drug-able (Table 4). Cochliophilin A, Quercimeritrin, and Miraxanthin III featured good molecular weight properties, which are all <500 Da, with log *P*-values of <5 indicating that they possess good lipophilicity. This implies that these phytocompounds are likely going to be membrane-permeable and would be orally absorbed. According to the Lipinski rule, a molecule will not be orally available if it violates two or more of the rules. Cochliophilin A and Miraxanthin III meet the requirements for oral bioavailability. Based on these molecular and physicochemical properties of the phytocompounds including log *P*, molecular reactivity, number of H-bonds acceptor, and donors, they possess features that make them drug-able. Cochliophilin A and Miraxanthin III likewise pass Veber’s rule of having 10 or fewer rotatable bonds and polar surface area (TPSA) not >140 Å^2^ (Veber *et al.* 2002).

Cochliophilin A has a 0.55 bioavailability score indicating that it is likely to be good as an oral drug. Pgp is an important member of the ATP-binding cassette transporters and it is responsible for the active efflux of xenobiotics through biological membranes to protect the body from foreign chemicals. It also contributes to drug resistance by limiting the entry of some drugs into sensitive areas. Cochliophilin A and Miraxanthin III are not substrates of Pgp, thus are not likely to be prevented from entering their target site of action. From the pharmacokinetic predictions, Cochliophilin A as a CYP inhibitor could cause drug–drug interaction, CYP superfamily of isoenzyme is the key phase I metabolism enzyme catalyst. However, Miraxanthin III did not display inhibitory potential against the various CYP isoenzymes (CYP 1A2, CYP 3A4, CYP 2C9, CYP 2C19, and CYP 2D6) and may not adversely affect Phase I drug metabolism in the liver. Toxicity prediction showed that both Cochliophilin A and Miraxanthin III were not hepatotoxic, mutagenic, and cytotoxic although Cochliophilin A was predicted to be slightly carcinogenic.

## 4 Conclusion

We evaluated the inhibitory effect of phytochemicals derived from beetroot against ACE and AR in hypertension. A total of 70 compounds reported to be present in beetroot were subjected to virtual screening, molecular docking, and MDSs. The three hit compounds—Cochliophilin A, Quercimeritrin, and Miraxanthin III, were selected due to the exhibition of high binding affinity in structure-based virtual screening. They had good ADMET profiles and the results for the MD trajectories showed that they formed strong hydrogen bonds with active site residues and stable complexes with the target proteins. Throughout the all-atom 100 ns MD simulation, the resulting trajectories of ACE and AR complexed with the lead phytocompounds showed higher stability and increased flexibility alongside higher interaction potential of the residues of the receptor toward the phytocompounds. SASA plots also indicated accessibility of the phytocompounds to the binding pockets. Based on available evidence, we present these phytocompounds as potential drug candidates for hypertension. Thus, reports from this study can enhance the development of safe nutraceuticals and drug candidates for the management or treatment of hypertension. Further experimental investigations and lead optimizations may help in optimizing these molecules for increased affinity and specificity for ACE and AR.
